# The Evolution and Future Directions of PBPK Modeling in FDA Regulatory Review

**DOI:** 10.3390/pharmaceutics17111413

**Published:** 2025-10-31

**Authors:** Yangkexin Li, Henry Sun, Zuoli Zhang

**Affiliations:** SunTech Research Institute, LLC., Rockville, MD 20850, USA

**Keywords:** physiologically based pharmacokinetic, PBPK model, drug–drug interaction (DDI), regulatory science, artificial intelligence

## Abstract

**Background:** Physiologically based pharmacokinetic (PBPK) modeling is a mathematical approach that integrates human physiological parameters with drug-specific characteristics (including both active pharmaceutical ingredients and excipients), and it has emerged as one of the core technologies for optimizing the efficiency and reliability of drug development. Methods: This study synthesizes applications of PBPK models in FDA-approved drugs (2020–2024), systematically analyzing model utilization frequency, indication distribution, application domains and choice of modeling platforms, to reveal their substantive contributions to regulatory submissions. Additionally, we conducted an in-depth analysis of the PBPK models for 2024, classifying models into three tiers based on critical assessment of FDA reviewer comments. **Results:** Among 245 FDA-approved new drugs during this period, 65 NDAs/BLAs (26.5%) submitted PBPK models as pivotal evidence. Oncology drugs accounted for the highest proportion (42%). In application scenarios, drug–drug interaction (DDI) was predominant (81.9%), followed by dose recommendations for patients with organ impairment (7.0%), pediatric population dosing prediction (2.6%), and food-effect evaluation. Regarding modeling platforms, Simcyp^®^ emerged as the industry-preferred modeling platform, with an 80% usage rate. In terms of regulatory evaluation, a core concern for reviewers is whether the model establishes a complete and credible chain of evidence from in vitro parameters to clinical predictions. **Conclusions:** Detailed regulatory reviews demonstrate that although some PBPK models exhibit certain limitations and shortcomings, this does not preclude them from demonstrating notable strengths and practical value in critical applications. Benefiting from the strong support these successful implementations provide for regulatory decision-making, the technology is gaining increasing recognition across the industry. Looking forward, the integration of PBPK modeling with artificial intelligence (AI) and multi-omics data will unprecedentedly enhance predictive accuracy, thereby providing critical and actionable insights for decision-making in precision medicine and global regulatory strategies.

## 1. Introduction

Physiologically based pharmacokinetic (PBPK) modeling is a mathematical framework that integrates human physiological and anatomical parameters with drug-specific physicochemical and biochemical properties to quantitatively predict pharmacokinetic (PK) profiles in specific tissues or human populations [1].

Conventional compartmental models, which are primarily data-fitting tools, conceptualize the body as a system of abstract mathematical compartments. The model parameters, obtained by fitting experimental plasma concentration-time data, lack direct physiological referents [2]. In contrast, PBPK modeling is structured upon a mechanism-driven paradigm, representing the body as a network of physiological compartments (e.g., liver, kidney, brain) interconnected by blood circulation, and integrating system-specific physiological parameters with drug-specific properties [3]. Such a mechanistic foundation provides PBPK modeling with remarkable extrapolation capability. For instance, in modeling absorption, these models can integrate parameters such as pH, permeability, and metabolic enzyme/transporter expression levels across different gastrointestinal segments to precisely simulate complex oral absorption behaviors. Regarding tissue distribution, they can estimate drug concentrations at target sites or potential organs of toxicity by defining tissue-specific partition coefficients, which is challenging to achieve with traditional approaches. Consequently, PBPK modeling is capable of not only describing observed pharmacokinetic data but also quantitatively predicting systemic and tissue-specific drug exposure under untested physiological or pathological conditions.

Leveraging these advantages, PBPK modeling demonstrates significant value across several critical phases of drug development [4,5]. In drug–drug interaction (DDI) assessments, this approach can dynamically and quantitatively predict the impact on substrate drug exposure by simulating the kinetics of metabolic enzyme or transporter inhibition/induction, thereby informing clinical risk management strategies for combination therapies [6,7]. In the realm of special population dosing, PBPK models virtualize pharmacokinetic profiles by incorporating population-specific physiological parameters. This approach is particularly valuable for simulating the altered drug exposure resulting from pathophysiological changes in key organs such as the gut and liver [8,9], as well as for accounting for the distinct physiological characteristics of pediatric, elderly, and pregnant populations [10,11,12]. Consequently, PBPK modeling provides crucial support for designing initial dosing regimens in groups where large-scale clinical trials are challenging. Furthermore, this technology is widely applied in areas such as formulation development and bioequivalence studies.

The growing adoption and application of PBPK models in both industry and academia have prompted regulatory agencies to establish formal guidelines for their use. In September 2018, the U.S. Food and Drug Administration (FDA) issued the industry guidance <Physiologically Based Pharmacokinetic Analyses-Format and Content>, formally recognizing the regulatory role of PBPK in drug–drug interaction (DDI) assessments [13]. In September 2020, the FDA expanded its framework by releasing the draft guidance <The Use of Physiologically Based Pharmacokinetic Analyses-Biopharmaceutics Applications for Oral Drug Product Development, Manufacturing Changes, and Controls>, which standardized PBPK applications in biopharmaceutics and offers sponsors detailed recommendations for incorporating PBPK analyses into investigational new drug submissions [14]. In December 2018, the European Medicines Agency (EMA) published its first PBPK-specific guideline, <Guideline on the Reporting of Physiologically Based Pharmacokinetic (PBPK) Modelling and Simulation>, marking the institutional recognition of this technology within the international regulatory framework [15].

Within this context, this study systematically analyzes the application of PBPK models in FDA-approved new drugs from 2020 to 2024. It evaluates the substantive contributions of PBPK models from multiple perspectives, including utilization frequency, therapeutic area distribution, application domains, choice of modeling platforms and qualitative analysis of regulatory acceptance. Based on these findings, we propose actionable recommendations on the future development direction of PBPK models in precision medicine and global regulatory strategies.

## 2. Methods

The following data-collection and analysis workflow was used:

### 2.1. Data Source and Exclusion Criteria

The study cohort included all novel drugs approved by the FDA’s Center for Drug Evaluation and Research (CDER) between January 2020 and December 2024. The drug list was extracted from the FDA’s annual <Advancing Health Through Innovation: New Drug Therapy Approvals> reports, which encompass New Molecular Entities (NMEs), new therapeutic biological products, and other drug products classified as novel therapies (e.g., new dosage forms, new combinations).

To precisely define the scope of our analysis around de novo drug development, we excluded the following submission types:

- Generic drugs and biosimilars, as the application of PBPK model in this context (e.g., bioequivalence waivers) constitutes a distinct, well-established regulatory paradigm not representative of its use in innovative drug development.
- Efficacy supplements for previously approved drugs, as these pertain to post-approval lifecycle management and fall outside the scope of this study on initial drug approval.

### 2.2. Identification of PBPK Applications

The approved-drug list was used to retrieve the complete review dossiers for each product from the Drugs@FDA database. Key documents were prioritized for screening: Multi-disciplinary review, Clinical pharmacology review, Clinical review and Integrated summary documents. A full-text search for the keywords “PBPK” and “physiologically based pharmacokinetic” was performed across these documents, followed by manual verification to confirm the presence and context of PBPK model application within the submission.

### 2.3. Data Extraction and Categorization

For each drug application utilizing PBPK model during 2020–2024, a standardized set of parameters was extracted. These included: Drug Name, Active Ingredient, Approval Date, Proposed Indication, Therapeutic Area, Modeling Platform (e.g., Simcyp, GastroPlus), Application Domain (e.g., drug–drug interaction [DDI], pediatrics, organ impairment [OI]), and Submission Type (NDA/BLA).

To capture the most contemporary regulatory perspective and leverage the complete review documentation available for the most recent approval cycles, a dedicated qualitative analysis was performed on the 2024 cohort. This involved extracting the FDA’s definitive assessment of the PBPK model’s utility, any identified model gaps or limitations, and verbatim quotations from the reviews to contextualize the regulatory perspective.

## 3. Results

### 3.1. PBPK Application Trends

**Figure 1:**
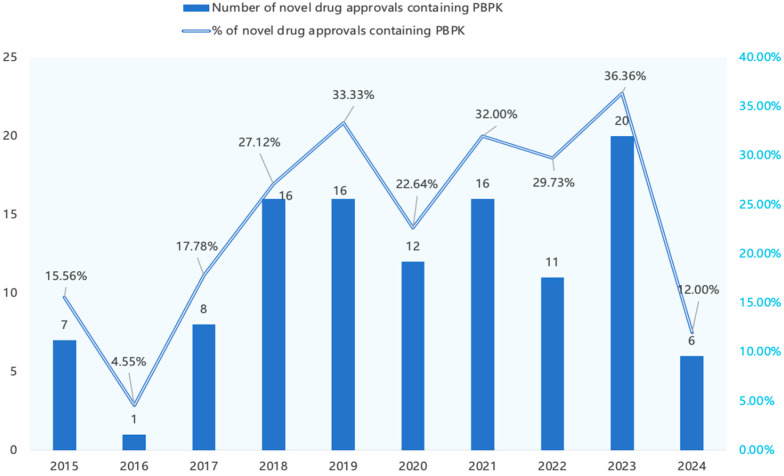
PBPK Model Use in FDA-Approved New Drugs (2015–2024).

Between 2020 and 2024, the FDA approved 245 new drugs, 65 NDAs/BLAs (26.5%) submitted PBPK models as pivotal evidence. To place our data within a historical context, we incorporated published data from Grimstein, Sun, Zhang et al. [5,16,17], which analyzed PBPK model applications in NDA documents between 2015 and 2023 using a similar research approach. Presenting this integrated data in Figure 1 reveals the following trends from 2015 to 2024: from 2015 to 2019, over 20% of new drug approvals incorporated PBPK models, although this proportion fell to 4.55% in 2016. The percentage increased to around 27% in 2018 and reached 33% in 2019. From 2020 to 2023, the proportion of approved new drugs using PBPK steadily exceeded 20%, but it decreased to 12% in 2024.

The bar chart shows the number of new drug approvals with PBPK models, and the line chart indicates the trend in the percentage of new drug approvals with PBPK models.

### 3.2. Therapeutic Area Distribution

**Figure 2:**
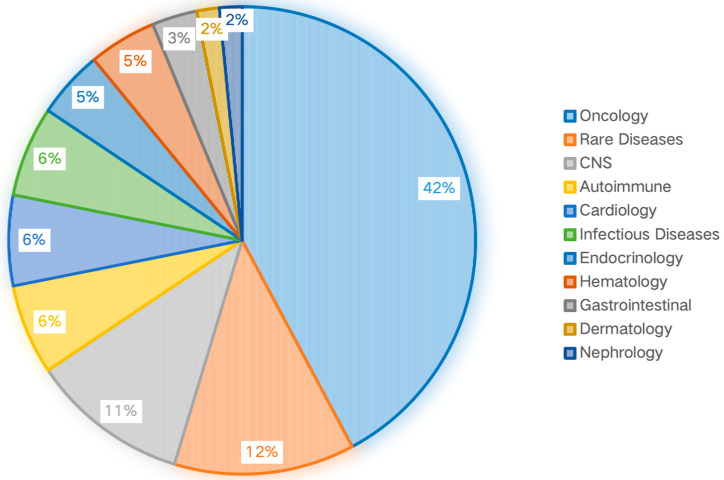
Distribution of Therapeutic Areas (2020–2024).

The statistical analysis of the submitted documents, categorized by therapeutic areas, indicates that oncology has the highest number (42%) of submissions, followed by Rare Diseases (12%), CNS (11%), Autoimmune (6%), Cardiology (6%) and Infectious Diseases (6%) (Figure 2).

### 3.3. Application Domains

Comprehensive analysis of the 65 drug submissions supported by PBPK models identified a total of 116 distinct application instances, as a single submission frequently employed models for multiple regulatory queries.

**Figure 3:**
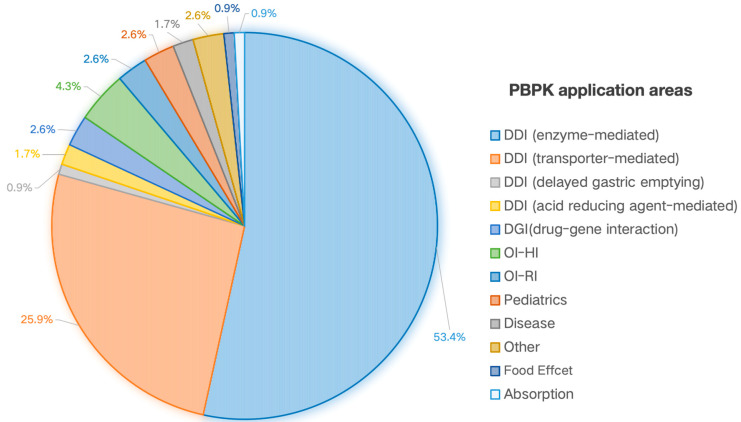
PBPK Model Application Domains in FDA-Approved New Drugs (2020–2024).

As delineated in Figure 3, the quantitative prediction of DDIs constituted the predominant application, representing 81.9% (95 of 116) of all instances. A granular breakdown of DDI mechanisms revealed that enzyme-mediated interactions (e.g., primarily CYP3A4) accounted for the majority (53.4%), followed by transporter-mediated interactions (e.g., P-gp, 25.9%). The remaining DDI instances involved acid-reducing agent-mediated interactions (1.7%) and simulations of delayed gastric emptying (0.9%).

Following DDI, PBPK modeling for guiding dosing in patients with organ impairment was the next most common application (7.0%), with specific use for hepatic impairment (4.3%) and renal impairment (2.6%). Applications for drug-gene interactions (DGI) and pediatric dosing strategies were equally represented, each constituting 2.6% of the total. Minor application domains included food-effect evaluation (0.9%) and absorption kinetics (0.9%). The “Other” category (2.6%) encompassed applications such as first-in-human PK prediction and tissue-specific concentration assessments.

### 3.4. PBPK Modeling Platform

**Figure 4:**
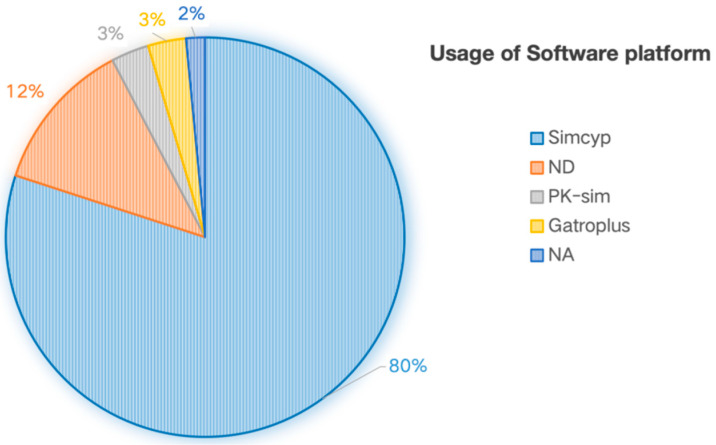
Distribution of PBPK Modeling Software Platforms (2020–2024).

According to this study’s statistical data, Simcyp^®^ platform dominated with an 80% adoption rate (Figure 4). In addition, 12% of submissions did not specify the modeling tool used. Notably, despite PK-Sim^®^ and GastroPlus^®^ demonstrating technical superiority in niche domains, their actual utilization rates in new drug applications remained at merely 3%, potentially indicating limited influence in innovative drug registration processes [18].

## 4. Case Studies

The case studies in this section are derived from a detailed assessment of the PBPK modeling sections within the complete FDA review documents for novel drugs approved in 2024. This cohort was selected to provide the most current perspective on regulatory modeling standards. The analysis revealed a spectrum of model acceptance, which we have synthesized and categorized into three distinct tiers based on the concluding assessments and explicit rationale provided by FDA reviewers. This taxonomy reflects the agency’s nuanced evaluation of a model’s utility in the specific regulatory context for which it was submitted.

Adequate: This classification is applied when the FDA’s review concludes that the model achieved its intended regulatory objective, was sufficiently verified, and provided direct support for a specific decision. This is often evidenced by the model’s output being used to justify dose selection, support a waiver for a clinical study (e.g., DDI), or inform specific language in the product label without requiring additional validation.

Adequate with Limitations: This category describes models that the FDA deemed fit-for-purpose for a specific application, but with explicitly stated caveats. Reviewer comments in these cases acknowledge the model’s utility while simultaneously detailing quantifiable scientific flaws, uncertainties, or restrictions in its application. The “limitations” are those formally recognized by the agency, which may reduce the model’s weight in the overall evidentiary package or constrain its use to a narrow set of conditions.

Inadequate: This tier is reserved for models that reviewers concluded contained substantial scientific deficiencies, which rendered their predictions insufficient to support the proposed regulatory decision. These deficiencies, such as unverified key parameters, incorrect structural assumptions, or failed validation, formed the basis for the FDA’s explicit rationale to reject the model’s use for its intended purpose, often necessitating a traditional clinical study instead.

### 4.1. Adequate

LIVDELZI (seladelpar) is a peroxisome proliferator–activated receptor δ (PPARδ) agonist. On 14 August 2024, it was approved by the FDA for the treatment of primary biliary cholangitis (PBC) [19], including the management of pruritus. It is intended for adults without liver cirrhosis and those with compensated cirrhosis.

The applicant developed a PBPK model using Simcyp^®^ to simulate the effects of a strong CYP2C9 inhibitor (sulphaphenazole), strong (itraconazole) and moderate (erythromycin) CYP3A inhibitors, and a strong CYP2C8 inhibitor (gemfibrozil) on seladelpar exposure.

Regulatory assessment indicated that the PBPK model was sufficient to support CYP enzyme-mediated DDI risk assessment for seladelpar and could inform labeling strategies.

Specifically, concomitant administration with the strong CYP2C9 inhibitor sulphaphenazole was predicted to increase the area under the plasma concentration-time curve extrapolated to infinity (AUC_inf_) of seladelpar by 3.71-fold, representing an unacceptably high risk necessitating strict avoidance. In contrast, no clinically meaningful interactions were predicted with moderate CYP2C9, strong/moderate CYP3A, or strong CYP2C8 inhibitors, allowing co-administration under medical supervision.

Model evaluation showed good predictive performance for seladelpar PK following single 10-mg or multiple once-daily dosing, with predicted AUC (area under the curve) and C_max_ values within 0.8–1.25-fold of observed data in healthy subjects. Although clinical DDI data with potent CYP3A and CYP2C8 inhibitors were lacking, targeted sensitivity analyses demonstrated that extreme inhibition scenarios would increase seladelpar AUC_inf_ by no more than 1.84-fold (CYP3A) or 1.95-fold (CYP2C8), remaining below the two-fold threshold of clinical concern. These results supported the model’s adequacy for assessing CYP enzyme–mediated DDI risks.

By contrast, the model showed notable deficiencies in simulating transporter-mediated interactions. Although intestinal, hepatic, and renal transporter mechanisms (PerL, MechKiM) and kinetic parameters for BCRP and OATP1B1 were implemented, predicted interactions with cyclosporine and probenecid substantially underestimated observed effects. Clinically, probenecid co-administration increased seladelpar AUC_inf_ (GMR = 2.0) and C_max_ (GMR = 4.69), whereas the model predicted minimal changes (AUC_inf_ GMR = 1.04; C_max_ GMR = 1.09). Furthermore, the model only considered renal OAT inhibition and did not capture transporter effects during absorption and distribution. Clinical findings, including a reduced volume of distribution (from 127 L to 50 L) and a shortened T_max_, indicated that it failed to replicate nonlinear peak dynamics.

Reviewers concluded that the transporter inhibition framework was scientifically incomplete and recommended incorporating additional absorption and distribution mechanisms to improve prediction of C_max_ shifts. However, these limitations were not considered critical for the model’s intended use [20].

### 4.2. Adequate with Limitations

#### 4.2.1. LEQSELVI

LEQSELVI (deuruxolitinib) is an orally administered selective Janus kinase inhibitor (JAK1/JAK2/TYK2 > JAK3) indicated for moderate-to-severe alopecia areata in adults, approved by the FDA on 25 July 2024 [21]. 

The applicant developed a PBPK model using Simcyp^®^ to evaluate complex DDI risks when deuruxolitinib is co-administered with strong CYP2C9 inhibitors (sulphaphenazole) or moderate CYP3A4 inhibitors/inducers (efavirenz), with a specific focus on individuals harboring different CYP2C9 genotypes (particularly in *CYP2C9*3/*3* poor metabolizers).

Regulatory assessment concluded that the model was suitable for guiding dose adjustment strategies and was used to update the drug label for patients with varying CYP2C9 genotypes when combined with CYP3A4 and CYP2C9 inhibitors/inducers.

However, this successful case was accompanied by inherent methodological limitations and trade-offs.

The model’s key adaptation was its departure from the in vitro reaction phenotyping study. That study had identified CYP3A4 as the primary metabolizing enzyme for deuruxolitinib, with a fractional metabolism (f_m_) of ~87%. However, a clinical DDI study showed only a weak interaction with the CYP3A4 inhibitor itraconazole (AUC ratio = 1.27). Consequently, the applicant recalibrated the in vivo enzyme contributions. CYP2C9 was instead designated as the primary enzyme (f_m_ ~ 76%), while the contribution of CYP3A4 was reduced to 21%. This adjustment enabled the model to accurately recapitulate several clinical observations and successfully predict the high-risk interaction with the strong CYP2C9 inhibitor sulphaphenazole (3-fold AUC increase), as well as the doubled baseline exposure in CYP2C9 poor metabolizers. Nevertheless, this correction was essentially an empirical calibration based on clinical data. It failed to mechanistically explain the significant disparity between the in vitro and in vivo findings. The exclusion of CYP2C19’s role also lacked direct confirmation, constituting a fundamental uncertainty in the model’s metabolic pathway parameters. Furthermore, the model employed a simplification in simulating CYP2C9*3/*3 poor metabolizers by assigning a fixed enzyme abundance, which may not fully capture the true variability within this population. Additionally, the empirical adjustment of absorption parameters to reconcile data from different formulations (powder-in-capsule, uncoated, and coated tablets) amounted to “curve-fitting,” deviating from the principles of mechanism-based modeling and undermining the model’s predictive capability for absorption processes. Although sensitivity analysis suggested that DDI predictions were insensitive to this adjustment, it remained a theoretical weakness. Finally, the model primarily focused on deuruxolitinib as a “victim” drug and did not systematically evaluate its potential as a “perpetrator” in DDIs. Consequently, the model’s predictive power was demonstrated more in its “retrospective” description of existing data than in its “prospective” prediction of more complex, unknown scenarios.

Overall, the deuruxolitinib PBPK model demonstrates a practical approach. Its utility resides in extrapolative predictions based on clinical data calibration, which quantified complex genotype-dependent DDI risks and provided critical regulatory decision support. However, it does not offer a definitive elucidation of the drug’s disposition mechanisms [22].

#### 4.2.2. VORANIGO

VORANIGO (vorasidenib) is an IDH1/IDH2 inhibitor approved by the FDA on 6 August 2024 for adult and pediatric patients 12 years and older with surgically intervened Grade 2 astrocytoma/oligodendroglioma harboring IDH1/IDH2 mutations [23].

The applicant developed a PBPK model using the Simcyp^®^ platform to predict DDI for vorasidenib in two contexts: as a victim drug during co-administration with CYP1A2 inhibitor fluvoxamine or inducers rifampin/phenytoin, and as a perpetrator drug affecting substrates of CYP3A4/2C8/2B6 enzymes and BCRP/P-gp transporters.

Regulatory assessments concluded that the model was deemed adequate to support labeling for vorasidenib as a “victim” drug, accurately predicting a 10-fold exposure increase with the strong CYP1A2 inhibitor fluvoxamine (contraindicated) and an ~40% decrease with moderate inducers (rifampin, phenytoin). Conversely, it was considered not adequate for predicting vorasidenib as a “perpetrator” of CYP3A4 and CYP2C8 induction, leading to a conservative label statement that a “DDI potential cannot be excluded.”, thereby alerting clinicians to potential interaction risks. Additionally, it could not be used to assess effects on the CYP2B6 substrate bupropion.

The PBPK model incorporated two distinct formulations: an early crystalline form (F1) and the optimized commercial amorphous solid dispersion (F2). It successfully characterized vorasidenib PK for both formulations at the 50 mg dose across healthy subjects and patients, with predictions within the 0.8–1.25-fold acceptance range.

Despite its strengths, the model’s limitations delineate the boundaries of its application; its shortcomings include uncertainty in induction parameter extrapolation, dependency on component models and the challenge of complex absorption mechanisms.

The primary shortfall was its inability to accurately predict enzyme induction, stemming from significant uncertainties in in vitro-to-in vivo extrapolation (IVIVE) of induction parameters. The clinical calibration of the CYP3A4 Indmax parameter was insufficient to validate the overall predictive framework for untested scenarios.

The model’s application was restricted to bupropion (a CYP2B6 substrate) because the underlying substrate model within the Simcyp platform itself was unvalidated. This highlights a systemic vulnerability in PBPK strategies, where the final output’s credibility is contingent on all constituent models.

The model underpredicted exposure at higher doses (100–300 mg) of the F1 formulation, indicating limitations of its mechanistic absorption model in handling the dissolution dynamics of a low-solubility drug with specific formulation characteristics [24].

Overall, the vorasidenib PBPK model exemplifies a qualified regulatory success. It successfully supported critical “victim” DDI assessments in the product label, while its clearly acknowledged limitations—particularly regarding induction—definitively shaped the drug’s safety communication and underscored the necessity for clinical caution in areas where model-based prediction remained insufficient.

#### 4.2.3. ALYFTREK

ALYFTREK is a fixed-dose combination of tezacaftor, vanzacaftor, and deutivacaftor approved by the FDA on 20 December 2024 for cystic fibrosis patients aged ≥6 years with F508del mutations or other responsive mutations [25]. The applicant developed three PBPK models (VNZ, D-IVA, and FDC) using the Simcyp^®^ platform to support combination therapy decisions. These models aimed to evaluate effects of moderate/strong CYP3A inhibitors/inducers on VNZ and D-IVA exposure in adults and children aged 6 through 11 years, establish CYP3A inhibitor-driven dose adjustments and assess perpetrator risk of VNZ/D-IVA against CYP2C9 substrates. Regulatory assessment concluded that while adult DDI predictions and dosing regimens were successfully validated, pediatric extrapolation proved inadequate and CYP2C9-mediated perpetrator risk remained incompletely resolved.

Regarding the VNZ model, adult single/multiple-dose DDI predictions demonstrated acceptable accuracy (predicted/observed ratios: 0.8–1.25). The strong CYP3A inhibitor regimen (once-weekly dosing) maintained steady-state AUC within 1.5-fold of baseline. However, systematic prediction bias emerged in pediatric subgroups. Children weighing <40 kg (typically corresponding to an age range of 6–11 years) exhibited a predicted/observed ratio of 1.32, attributable to unmodeled age-dependent CYP3A4 maturation. High variability in clinical verification data (CV > 50%) further compromised extrapolation reliability.

Regarding the D-IVA model, basic validation passed with <15% prediction error for single-dose itraconazole DDI, and predictions were acceptable for children ≥40 kg. However, three critical limitations persisted. First, predictive accuracy significantly decreased in the <40 kg group (predicted/observed ratio: 1.5). Second, the absence of IC_50_ sensitivity analysis precluded quantification of risk scenarios, including a 3-fold IC_50_ reduction causing a 25% tolbutamide AUC increase above thresholds. Third, the omission of the inhibitory potential of metabolite M1-D-IVA (IC_50_ = 4.6 µM) compromised risk assessment completeness.

The integrated application model defined fixed-dose combination regimens involving weekly administration with strong CYP3A inhibitors and alternate-day dosing with moderate inhibitors. It contraindicated concomitant strong/moderate CYP3A inducers (e.g., rifampin reduced VNZ exposure by 82%). Core limitations included pediatric dosing directly adopting adult DDI ratios without correcting established biases, unquantified inducer effects on metabolite clearance and inadequate CYP2C9 risk management limited to “cautious use” recommendations without IC_50_-threshold-based monitoring protocols.

Consequently, the FDA issued an “Adequate with Major Limitations” regulatory decision for this PBPK submission, mandating three binding requirements. A black box warning was implemented stating “Dosing for patients under 40 kg is based on unvalidated PBPK models”. Post-marketing clinical verification studies were ordered to evaluate D-IVA’s IC_50_ under 3-fold reduction scenarios. Supplemental labeling requirements were imposed for drug interactions sections, specifying that “CYP2C9 inhibition potential of metabolite M1-D-IVA remains incompletely characterized” [26].

### 4.3. Inadequate

#### 4.3.1. ATTRUBY

ATTRUBY (acoramidis) is a transthyretin stabilizer approved by the FDA on 22 November 2024 for treating wild-type or hereditary transthyretin-mediated amyloid cardiomyopathy (ATTR-CM) in adults to reduce cardiovascular mortality and hospitalization risks [27].

The applicant developed a PBPK model using Simcyp^®^ to evaluate acoramidis both as a “perpetrator” of CYP2C8/9-mediated DDIs (e.g., with substrates like repaglinide for CYP2C8, and tolbutamide or warfarin for CYP2C9) and as a “victim” of UDP-glucuronosyltransferases (UGT) enzyme induction (e.g., by rifampin).

Regulatory assessments concluded that the model was inadequate to support regulatory decision-making. Specifically, for its role as a CYP2C9 perpetrator, the model was deemed insufficient to waive a clinical DDI study, and reviewers recommended that a clinical DDI study be conducted directly. For UGT induction, the quantitative predictions were considered unreliable and were recommended for further investigation.

The model’s inadequacy stemmed from several critical limitations: lack of clinical verification for metabolic pathways; unsubstantiated assumptions in UGT induction; lack of predictive robustness and clinical confirmation; and non-mechanistic parameter adjustments.

A primary weakness was the lack of clinical verification for its metabolic pathways. The assumptions that UGT metabolism contributed approximately 30% to clearance and the quantitative contributions of specific UGT isoforms (1A1, 1A9, 2B7) were derived solely from in vitro and ADME studies, without confirmation from clinical DDI data. This uncertainty not only undermined the predictions of acoramidis as a victim drug but also implied that its inhibition potential as a perpetrator might be incomplete due to unknown clearance pathways.

Compounding this issue were unsubstantiated assumptions in UGT induction. The model assumed all relevant UGT isoforms shared identical induction parameters derived from UGT1A1, overlooking potential differences in regulatory mechanisms (e.g., PXR, CAR) and induction sensitivity among isoforms. Moreover, extrapolating rifampin’s induction parameters to UGT1A9 and UGT2B7 lacked clinical data support, and the absence of data on the effects of other strong inducers (e.g., carbamazepine, phenytoin) on the UGT pathway. This limited the ability to assess the maximum induction potential and further undermines the reliability of the predictions.

Beyond these flaws, the model demonstrated a critical lack of predictive robustness and clinical confirmation. Sensitivity analysis revealed that minor adjustments to the in vitro inhibition parameter (K_i_), such as a 10-fold decrease, could escalate the predicted DDI for CYP2C9 substrates from >2-fold to >5-fold. This high sensitivity indicated that the predictions heavily relied on a single, potentially inaccurate in vitro measurement, failing the test of robustness. It was this unacceptable level of uncertainty that directly prompted reviewers to mandate a clinical DDI study, demonstrating that the model itself was insufficient to independently inform dosing decisions related to CYP2C9.

Finally, the model’s scientific rigor was further compromised by non-mechanistic parameter adjustments. To reconcile single and multiple-dose PK data, the applicant empirically assigned two different clearance values without a mechanistic rationale. This “curve-fitting” approach fundamentally contravened the principle of mechanistic modeling in PBPK, thereby compromising its scientific rigor [28].

In summary, the acoramidis PBPK model was fundamentally built on unverified inferences and assumptions. Throughout the review, reviewers consistently emphasized the pervasive uncertainty. Therefore, the model is inadequate.

#### 4.3.2. ITOVEBI

ITOVEBI (Inavolisib) is a small molecule kinase inhibitor of the phosphatidylinositol 3-kinase (PI3K) with inhibitory activity predominantly against PI3Kα. On 10 October 2024, inavolisib was approved in combination with palbociclib and fulvestrant by FDA for treating adult patients with hormone receptor (HR)-positive, human epidermal growth factor receptor 2 (HER2)-negative, locally advanced or metastatic breast cancer harboring PIK3CA mutations and endocrine therapy resistance [29].

The applicant developed a PBPK model using Simcyp^®^ to predict the net drug–drug interaction (DDI) effect of inavolisib on CYP3A substrates, a particular challenge as inavolisib acts as a mixed perpetrator, exhibiting both time-dependent inhibition and induction of CYP3A in vitro.

Regulatory assessment concluded that the model failed to quantify the induction risk for sensitive CYP3A substrates like midazolam. This failure is fundamentally rooted in the intrinsic inability of the current modeling approach to decouple the opposing inhibition and induction mechanisms and confidently predict the net clinical outcome.

The methodological deficiencies were profound. The model relied on an obsolete “rifampicin-based in vitro calibration approach” to derive induction parameters, despite regulatory precedence that robust CYP3A induction models require clinical DDI data for calibration. This approach is particularly inadequate for a mixed perpetrator like inavolisib, as it cannot resolve the competing dynamics of concurrent enzyme inactivation and synthesis.

Underpinning these methodological flaws were critical data reliability issues. The hepatocyte induction data from three donors was compromised by drug crystallization at high concentrations and a failure to achieve maximum induction response, leading to underestimated E_max_ values. Furthermore, the selection of data from only one donor (Lot FOS) without biological justification introduced unquantifiable bias.

Finally, the model verification constituted a circular argument. Using palbociclib—a non-sensitive CYP3A substrate cleared by multiple pathways as a validation tool was destined to fail. Reviewers demonstrated that even under maximal simulated induction scenarios that predicted a 50% AUC reduction for the sensitive substrate midazolam, the model showed no significant change in palbociclib exposure. This verification failure underscores the model’s fundamental lack of sensitivity to capture the very CYP3A induction effect it was intended to predict, a critical shortcoming for a drug with dual and opposing effects.

Consequently, regulatory agencies rejected the model’s predictive capability due to these compounded and interdependent deficiencies, ultimately mandating clinical DDI studies to resolve the uncertainty [30].

## 5. Discussion

### 5.1. Trend Analysis

Based on FDA review data from 2020 to 2024, PBPK models were incorporated in approximately 26% of new drug applications (NDAs/BLAs), demonstrating their emergence as a mainstream tool in drug development and regulatory decision-making. However, the utilization rate of PBPK models declined to 12% in 2024, suggesting its journey toward ubiquitous adoption is not linear. This fluctuation underscores a fundamental context-dependency in PBPK application, which may be attributable to evolving drug development portfolios, the maturation of modeling strategies, and shifting regulatory priorities.

The Therapeutic area distribution reveals that.

The distribution of PBPK applications across therapeutic areas is highly heterogeneous. Oncology dominates, accounting for 42% of submissions, which likely reflects the complex pharmacokinetics, narrow therapeutic index, and high prevalence of polypharmacy (and thus DDI risk) in this patient population [31]. This is followed by rare diseases (12%), central nervous system disorders (11%), and others (Figure 2). The concentration of PBPK use in these areas underscores its role in addressing specific, high-stakes development challenges, such as optimizing dosing in severe conditions or in populations where clinical trials are difficult [32].

The application domain analysis reveals that the PBPK model is most extensively applied in DDI assessments (81.9%), with its simulation capabilities receiving broad FDA recognition. However, this contrasts sharply with the limited adoption in special populations, such as patients with organ impairment (7.0%) and pediatrics (2.6%). This constrained application likely stems from the greater challenges in characterizing population-specific physiological variances and obtaining sufficient clinical data for model verification in these cohorts.

Regarding modeling platforms, the Simcyp^®^ Simulator dominated submissions, being employed in 80% of PBPK-supported applications. The limited adoption of platforms with niche technical advantages, such as GastroPlus^®^ (noted for its strength in predicting oral absorption, accurately simulating bioavailability for different release characteristics and providing key evidence for optimal dissolution and bioequivalence studies) and PK-Sim^®^ (valued for its open-source nature) [33,34], which collectively accounted for only 3% of cases, may reflect broader industry trends in tool standardization rather than deficiencies in the platforms themselves. Notably, version inconsistencies in Simcyp^®^ implementations across FDA drug review dossiers highlight an industry-wide challenge in model standardization and version alignment, warranting explicit regulatory guidance to ensure methodological consistency.

### 5.2. Case Study Synthesis

Our detailed assessment of the PBPK modeling sections within the complete FDA review documents for novel drugs approved in 2024 reveals that even models deemed sufficient for regulatory action often present identifiable limitations. These commonly include suboptimal structural design, unreliable sourcing of critical parameters, insufficient verification (both internal and external), and inadequate sensitivity analysis, etc.

After summarizing, we found that the three core factors that regulators use to evaluate the credibility of a model are whether the parameters are reliable, whether direct clinical verification is conducted, and whether the chain of evidence is complete and closed.

The credibility of any PBPK model is contingent upon the quality of its input parameters. This requires that critical values, such as enzyme contribution fractions (f_m_) and inhibition constants (K_i_), are sourced from robust, well-designed experiments rather than unverified assumptions. The case of LIVDELZI (seladelpar) exemplifies success, with its enzyme f_m_ values grounded in comprehensive in vitro data. In stark contrast, the failure of ATTRUBY (acoramidis) was precipitated by a model whose DDI predictions exhibited critical sensitivity to a single, unvalidated in vitro K_i_ value, exposing a fundamental lack of robustness.

Second, direct clinical verification is paramount. The regulatory confidence in a PBPK model’s prediction is greatest when it is directly verified against a dedicated clinical DDI study of the specific interaction in question. The strength of evidence diminishes with indirect approaches, such as extrapolation from other inhibitors or purely theoretical reasoning.

This evidentiary hierarchy is clearly demonstrated by our cases: The LIVDELZI (seladelpar) model achieved the “Adequate” because it was directly calibrated and verified against clinical data from a study with a strong CYP2C9 inhibitor. In contrast, the LEQSELVI (deuruxolitinib) model relied on indirect inference and extrapolation from other clinical data, which introduced substantial uncertainty and resulted in its classification as “Adequate with Limitations”.

An “Adequate” model must form a coherent and self-consistent narrative that seamlessly connects in vitro data to clinical observations and, finally, to the intended regulatory prediction. LIVDELZI exemplifies this ideal, with a coherent story from in vitro f_m_ to clinical DDI calibration and finally prospective prediction. Conversely, ATTRUBY’s evidence chain was fractured between its in vitro assumptions and the in vivo reality, while LEQSELVI’s chain contained a weakened link in its chain due to non-mechanistic, empirical adjustments. In short, any deficiency in the chain of evidence will make the entire model unreliable.

In conclusion, the regulatory endorsement of a PBPK model is fundamentally an endorsement of the quality, directness, and integration of its constituent clinical pharmacology evidence.

### 5.3. Challenges and Limitations

While PBPK modeling has secured its role in regulatory submissions, our case-based analysis reveals persistent challenges that constrain its broader application and predictive confidence. These limitations, frequently cited in FDA reviews and evident across our case studies, can be categorized into two critical areas: insufficient parameter precision, oversimplification of model structures and inadequate mechanistic representation. Furthermore, beyond these case model-specific issues, the technology continues to grapple with the lack of standardized performance criteria and dedicated databases for special populations.

#### 5.3.1. Insufficient Parameter Precision

The credibility of any PBPK model is fundamentally dependent on the precision of its system-specific and drug-specific parameters [35]. These inputs are influenced by population demographics, in vitro data quality, and the robustness of in vitro to in vivo extrapolation (IVIVE). Interindividual physiological variability further complicates this landscape, necessitating precise alignment between simulated and actual population characteristics to improve both mean PK parameter estimation and variability characterization [36].

Crucially, our case studies demonstrate that parameter uncertainty is not a theoretical concern but a primary reason for limited regulatory acceptance. For instance, the ATTRUBY model’s inadequacy was directly attributable to its over-reliance on a single, unverified in vitro K_i_ value, which led to non-robust DDI predictions. The LEQSELVI model, while ultimately accepted, required non-mechanistic, empirical adjustments to key parameters (e.g., enzyme contribution fractions) to reconcile in vitro and clinical data, highlighting a fundamental uncertainty in its foundational parameters.

These examples underscore that the inability to obtain reliable parameter values due to insufficient experimental data or physiological complexity remains the most common PBPK-related issue faced by regulators, often leading to models being classified as “Adequate with Limitations” or “Inadequate”.

#### 5.3.2. Oversimplified Model Structures and Inadequate Mechanistic Representation

Another pivotal challenge identified in our case studies is the oversimplification of model structures and the inadequate representation of underlying biological mechanisms. This often manifests as the use of empirical, non-mechanistic adjustments to force model outputs to align with observed clinical data, thereby compromising the core “mechanism-driven” principle of PBPK modeling and its utility for prospective prediction.

The LEQSELVI model serves as a prime example. The model deviated from the initial in vitro reaction phenotyping results, which identified CYP3A4 as the primary enzyme, and instead empirically re-calibrated the in vivo enzyme contributions (designating CYP2C9 as primary) based on clinical DDI observations. While this adjustment enabled the model to recapitulate specific clinical datasets, it failed to provide a mechanistic explanation for the significant disparity between in vitro and in vivo findings. Furthermore, the model employed empirical “curve-fitting” of absorption parameters to reconcile data from different formulations. These approaches essentially traded the model’s prospective predictive power for a superior retrospective description of existing data. When a model’s structure is not grounded in verifiable physiology and biochemistry, its reliability for forecasting drug behavior in untested scenarios remains fundamentally limited.

#### 5.3.3. Lack of Validation Standardization

Furthermore, the field lacks standardized and quantitative criteria for model validation. Despite the FDA and EMA having issued technical guidance on PBPK models, international consensus remains absent regarding validation protocols, such as standardized clinical data verification ratios and sensitivity analysis thresholds. In most submissions, model performance evaluations predominantly rely on subjective assessments. The International Consortium for Innovation and Quality (IQ) PBPK Working Group advocates a methodological framework mandating pre-implementation verification of model compliance with predefined fit-for-purpose criteria [37].

#### 5.3.4. Cross-Ethnic Data Deficiencies

Another fundamental limitation is the lack of diverse, cross-ethnic physiological data in model parameter libraries. Core parameter libraries in mainstream platforms (e.g., Simcyp^®^, GastroPlus^®^) including hepatic enzyme activities and tissue perfusion rates that are predominantly constructed from population data of single geographic or ethnic origins [38]. Assumptions based on such ethnically specific physiological features significantly weaken the predictive accuracy of models in global drug development.

FDA data indicate minimal use of cross-ethnic parameters in PBPK-supported submissions (2020–2024). Systematic integration of cross-population data could revolutionize personalized medicine. However, this paradigm shift demands transition from retrospective literature mining to prospective real-world evidence generation which will bring huge and substantial technical challenges.

### 5.4. Future Directions

The future evolution of PBPK modeling will be crucial for overcoming the current limitations detailed in this analysis and for solidifying its role in predictive regulatory science. Directions worthy of attention and expansion may include the combination of artificial intelligence and machine learning, the integration with other modeling methods, and the combination with in vivo mechanism data, etc.

#### 5.4.1. AI Integration

Artificial Intelligence (AI) and Machine Learning (ML) algorithms hold immense potential to address the critical challenge of parameter precision and identification.

AI originated from the pursuit of machines emulating human intelligence, tracing back to seminal concepts proposed by pioneers like Alan Turing [39]. Its core objective is to endow computer systems with capabilities for perception, comprehension, learning, reasoning, and decision-making that parallel human cognition.

ML is a pivotal subfield and methodology of AI, which focuses on developing algorithms that autonomously learn and improve from data. This encompasses key learning modalities: supervised, unsupervised, semi-supervised, and reinforcement learning. These paradigms dictate how AI systems extract knowledge and optimize performance from data.

Traditional PBPK models, reliant on expert-driven structural construction, parameter calibration, and sensitivity analysis, face inherent limitations in efficiency and objectivity.

ML algorithms leverage large-scale datasets (e.g., compound physicochemical properties, transporter expression, enzyme metabolic profiles) to significantly enhance parameter prediction efficiency and accuracy. They can assist PBPK model structural optimization, virtual population generation, and improvements in model interpretability and predictive precision [40,41].

As a key ML subset, deep learning (DL) employs artificial neural networks to extract critical features from high-dimensional unstructured data [42]. This capability can be used to identify the complex nonlinear parameter relationships in the PBPK model and correct parameter deviations.

Reinforcement Learning (RL), as an algorithm adept at achieving the maximum benefit in an environment, is highly proficient in optimizing decisions [43]. It can find the optimal solutions for parameters and can be used to assist PBPK models in model calibration and dose plan optimization.

Generative adversarial networks (GANs) comprise two adversarial models: a generator and a discriminator. These two models engage in a competitive relationship, constantly adjusting their parameters. The ultimate goal is to make the discriminator unable to determine whether the output of the generator is genuine [44]. It can assist the PBPK model in generating synthetic data to expand the training set, generating new correction strategies, and optimizing the model’s capabilities. In scenarios involving the medication of specific populations, it can assist in filling in data gaps, thereby enhancing the extrapolation ability of the PBPK model.

In summary, the incorporation of AI technology will undoubtedly significantly enhance the modeling ability of the PBPK model. Specifically, in terms of parameter prediction, structure optimization, and data extrapolation, it will significantly improve the accuracy and reliability of the model, laying the foundation for more precise PK predictions.

#### 5.4.2. Hybrid Modeling

Integrating PBPK with complementary pharmacometrics approaches is revolutionizing quantitative pharmacology. Coupling with population PK (PopPK) models enriches physiological frameworks with real-world variability data, improving predictions for special populations (pediatric, hepatic/renal impairment) [45]. Synergy with PD models addresses limitations of plasma concentration-driven PK/PD approaches by simulating tissue-specific drug distributions. This enables precise dose–response characterization using target-organ concentrations rather than systemic exposure, enhancing efficacy/toxicity predictions and supporting personalized dosing [46].

#### 5.4.3. Mechanistic Data Fusion

Multi-omics technologies are catalyzing PBPK’s transition from “homogeneous virtual humans” to “personalized digital humans.” Convergence with systems biology, transcriptomics, and metabolomics will propel PBPK into high-dimensional systems pharmacology. Traditional models often oversimplify pathological/genetic heterogeneity using population-average parameters. Single-cell sequencing and multi-omics now enable granular mapping of disease-specific enzyme/transporter expression, permitting patient-tailored PBPK models, particularly transformative for rare diseases, pediatrics, and organ impairment populations.

### 5.5. Study Limitations

While this review provides a comprehensive analysis of PBPK applications in FDA submissions, several limitations should be acknowledged. While this analysis offers insights into PBPK modeling within FDA submissions, its conclusions are subject to several limitations that should be considered.

First, the regulatory documents included in this study were sourced only from the US FDA. Although a pivotal regulatory agency, the FDA’s specific review practices and acceptance thresholds may not be fully representative of other major jurisdictions, such as the European Medicines Agency (EMA) or the Japanese Pharmaceuticals and Medical Devices Agency (PMDA). Therefore, the trends and case-based findings reported here are primarily contextual to the U.S. regulatory landscape. Second, the depth of our analysis was inherently constrained by its reliance on publicly available review documents. The unavailability of certain proprietary model parameters, comprehensive validation reports, and internal regulatory deliberations limited our ability to perform a more granular, technical critique of the submitted models.

These limitations delineate clear pathways for future research. Subsequent studies could expand the scope to enable a comparative analysis of PBPK acceptance criteria across different regulators.

## 6. Conclusions

In summary, the PBPK model is a mechanism-based extrapolation method that has demonstrated a substantial increase in both its application scope and depth. It has been increasingly adopted by sponsors and submitted to regulatory agencies as key evidence. In response, regulatory agencies have issued multiple draft guidance documents to standardize and promote the appropriate application of this technology.

This review indicates that although PBPK models demonstrate significant advantages and application value in domains such as DDI and dose selection for special populations, several limitations persist. These are primarily reflected in the imprecision of parameters, the incompleteness of validation, and the lack of standardization. Nonetheless, regulatory agencies generally maintain a positive and open attitude toward this technology, acknowledging its potential to fulfill specific predictive goals in specific scenarios, while also cautiously emphasizing that its predictive performance highly depends on the quality of input data and the robustness of model verification.

In the future, the incorporation of methods such as artificial intelligence will enhance the accuracy of the model and unify the verification standards, thereby further improving the prediction accuracy of PBPK models and providing more scientific decision-making support for precision medicine and global regulatory strategies.

## Data Availability

The author declares that all the data supporting the findings of this study are contained within the paper.

## Abbreviations

PBPK	Physiologically based pharmacokinetic
PK	pharmacokinetics
PD	pharmacodynamic
FDA	the U.S. Food and Drug Administration
DDI	drug–drug interaction
EMA	European Medicines Agency
NMEs	New Molecular Entities
NDA	New Drug Application
BLA	Biologics License Application
DGI	drug–gene interaction
OI	organ impairment
P-gp	P-glycoprotein
IVIVE	in vitro-in vivo extrapolation
AUC_inf_	area under the plasma concentration-time curve extrapolated to infinity
AUC	area under the curve
f_m_	fraction metabolized
UGT	UDP-glucuronosyltransferases
AI	artificial intelligence
ML	machine learning
RL	Reinforcement Learning
GANs	Generative adversarial networks
